# Tissue-Resident Memory T Cells in Gastrointestinal Cancers: Prognostic Significance and Therapeutic Implications

**DOI:** 10.3390/biomedicines12061342

**Published:** 2024-06-17

**Authors:** Hiromichi Sato, Sikun Meng, Tomoaki Hara, Yoshiko Tsuji, Yasuko Arao, Kazuki Sasaki, Shogo Kobayashi, Eric di Luccio, Takaaki Hirotsu, Taroh Satoh, Yuichiro Doki, Hidetoshi Eguchi, Hideshi Ishii

**Affiliations:** 1 Department of Medical Data Science, Center of Medical Innovation and Translational Research, Osaka University Graduate School of Medicine, Yamadaoka 2-2, Suita 565-0871, Japan; hiromichi7286@gmail.com (H.S.);; 2 Department of Gastroenterological Surgery, Osaka University Graduate School of Medicine, Yamadaoka 2-2, Suita 565-0871, Japan; 3 Hirotsu Bio Science Inc., Chiyoda-Ku, Tokyo 102-0094, Japan

**Keywords:** tissue-resident memory T cells, gastrointestinal cancers, prognostic implications, cancer immunotherapy, therapeutic strategies

## Abstract

Gastrointestinal cancers, which include a variety of esophageal and colorectal malignancies, present a global health challenge and require effective treatment strategies. In the evolving field of cancer immunotherapy, tissue-resident memory T cells (Trm cells) have emerged as important players in the immune response within nonlymphoid tissues. In this review, we summarize the characteristics and functions of Trm cells and discuss their profound implications for patient outcomes in gastrointestinal cancers. Positioned strategically in peripheral tissues, Trm cells have functions beyond immune surveillance, affecting tumor progression, prognosis, and response to immunotherapy. Studies indicate that Trm cells are prognostic markers and correlate positively with enhanced survival. Their presence in the tumor microenvironment has sparked interest in their therapeutic potential, particularly with respect to immune checkpoint inhibitors, which may improve cancer treatment. Understanding how Trm cells work will not only help to prevent cancer spread through effective treatment but will also contribute to disease prevention at early stages as well as vaccine development. The role of Trm cells goes beyond just cancer, and they have potential applications in infectious and autoimmune diseases. This review provides a thorough analysis of Trm cells in gastrointestinal cancers, which may lead to personalized and effective cancer therapies.

## 1. Introduction

Gastrointestinal cancers, such as pancreatic cancer [1] and colorectal cancer [2] (CRC), remain formidable medical challenges with a substantial effect on global public health. The rising incidence and often late-stage diagnosis of these malignancies underscore the urgency of developing effective treatment strategies. Recently, cancer immunotherapy has shown remarkable progress in the treatment of various cancers; however, the role of tissue-resident memory T cells (Trm cells) in gastrointestinal cancers has become a subject of increased interest [3,4,5,6].

**Figure 1:**
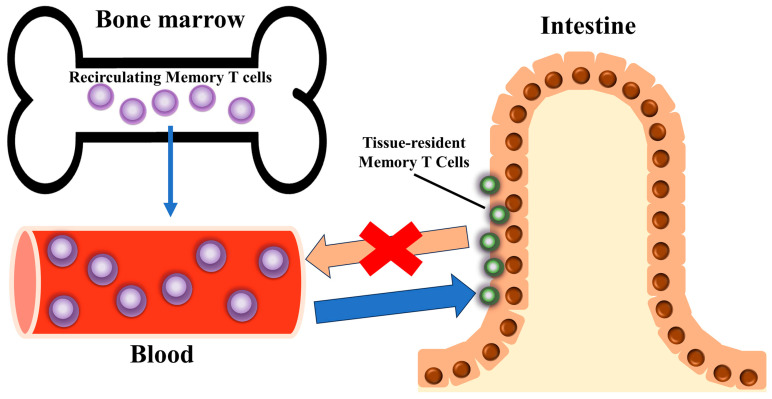
Tissue-resident memory T cell (Trm cell) localization. Characteristically, they reside in specific tissues and organs, effectively modulate the immune response in those tissues, and do not recirculate into blood vessels.

Trm cells, initially described as a specialized subset of memory T cells, are uniquely located within nonlymphoid tissues, where they provide rapid and localized immune responses toward pathogens and malignancy [3,7,8,9]. They are primarily found in peripheral tissues, do not recirculate in the blood, and elicit a rapid immune response in the initial stages of pathogen invasion [4] (Figure 1). Trm cells are present in various solid tumors, correlate with an improved prognosis, and can defend against tumor attack in mice [10].

The uptake and metabolism of exogenous lipids play an important role in the maintenance, longevity, and function of CD8+ Trm cells [11] and trigger the initial response to infections re-encountered on the body surface, where the clearance of pathogens occurs [7,12,13]. In the early stages of acute viral and bacterial pathogeneses, intestinal antigen-specific Trm cells were identified, including the most prominent Blimp1hiId3lo tissue-resident effector cell population. They exhibit various cytokine production abilities, secondary memory capacity, and transcriptional programs, including different roles for the transcriptional regulators, Blimp1, T-bet, Id2, and Id3, to support and maintain gut Trm cells [14]. These cells provide robust immune protection against a wide breadth of both viral and bacterial infections and/or reinfection because of their presence at sites of pathogen entry [15]. Active Trm cell responses also contribute to the development of various chronic respiratory diseases, including pulmonary sequelae following acute viral infection [16]. The phenotypic, transcriptional, and functional characteristics of Trm cells have been elucidated previously in mouse models of infection [17].

Trm cells also play an important role in the pathogenesis of host antifungal infections [18], antimicrobial infections, cancer immunotherapy, and various human autoimmune diseases, such as psoriasis [19,20], vitiligo [19,21], atopic dermatitis [22], lupus nephritis, antineutrophil cytoplasmic antibody-associated glomerulonephritis [23], rheumatoid arthritis [24,25], and inflammatory bowel disease [4]. In the perivascular lumen, cuffs containing CD8+ Trm cells have been observed in advanced multiple sclerosis, suggesting that they may be involved in local reactivation sites [26]. Trm cells may also contribute to autoimmune reactions during organ transplantation [27,28]. Combined single-cell RNA and T-cell receptor (TCR)-sequencing analyses of recipient-derived T cells from the bronchoalveolar lavage fluid of three recipients with lung transplant rejection revealed the accumulation of cytotoxic recipient-derived Trm cells within lung allografts, even after rejection treatment with high-dose systemic glucocorticoids [29].

**Figure 2:**
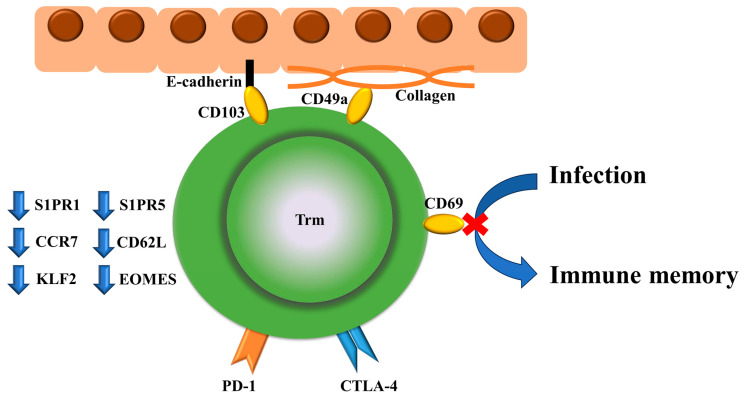
Surface markers specific to Trm cells and their functions, and markers that are downregulated are shown. Trm cells play a role in PD-1/CTLA-4-mediated cancer immunity, infection defense, and thereby, immune memory. CD103 and CD49a are associated with E-cadherin and collagen, respectively. Trm cells are characterized by the downregulation of S1PR1, S1PR5, CD62L, CCR7, KLF4, and eomesodermin (EOMES).

Trm cell function extends beyond immune surveillance including CD8+CD69+CD103+ Trm cells [30], which renders them key players in the intricate immune landscape of gastrointestinal cancer. The abundance of skin Trm cells, which are CD69- or CD103-positive cells, was increased in skin lesions of cutaneous lupus erythematosus, whereas interferon (IFN)-α increased CD69 expression in T cells [31]. CD103 is not just a biomarker for Trm cells but provides substrate specificity for cell adhesion to e-cadherin, and CD49a is a collagen-binding integrin [32,33]. In contrast, Trm cells are characterized by downregulation of S1PR1 [34,35,36], S1PR5 [37], CD62L [34], CCR7 [34], KLF4 [35], and eomesodermin (EOMES) [38] (Figure 2). These new findings associated with CD8+ T-cell immunity will lead to the development of more effective preventive and therapeutic interventions against cancer and infectious diseases [39]. A recent study indicated that ICOS-PI3K signaling promotes the establishment of CD8+ Trm cells [40]. Dendritic cell-presented antigens and interleukin (IL)-15 play important roles in antigen persistence and the maintenance of CD8+ Trm cells during inflammation [41].

Trm cells have been identified in the tumor microenvironment (TME) of pancreatic cancer [42] and CRC [4,43], which raises important questions regarding their contribution to antitumor immunity and potential for therapeutic manipulation. In this review, we discuss the emerging field of Trm cells in the context of gastrointestinal cancers. We aimed to comprehensively analyze the current knowledge on the role of Trm cells in these malignancies. We will examine their presence, functions, and potential effects on patient outcomes and discuss the prospects of exploiting Trm cells for novel treatment strategies. This analysis of the intersection of Trm cells and gastrointestinal cancers will contribute to a deeper understanding of the complex immunological dynamics that occur and stimulate further research in pursuit of more effective immunotherapies for cancer.

## 2. Trm Cells and Gastrointestinal Cancers

Trm cells have gained significant recognition as pivotal components of the immune response against various cancers, such as lung cancer [44,45]. In the intricate context of the gastrointestinal tract, particularly in pancreatic cancer [42] and CRC [43], the interactions between Trm cells and malignancies have taken center stage. In this section, we discuss the effect of Trm cells on gastrointestinal cancer and explore their role in tumor progression, patient prognosis, and as targets for therapeutic intervention.

### 2.1. Immune Surveillance by Trm Cells in the Gastrointestinal Tumor Microenvironment

The gastrointestinal TME provides a unique site for the interaction between Trm and cancer cells. The location of Trm cells in pancreatic cancer and CRC tissues has captured the attention of researchers and prompted studies regarding their spatial distribution and abundance. This important finding provides insights into the extent of Trm cell infiltration within these tumors.

Trm cells are known for their role in immune surveillance within tissues, and immune surveillance by cells with a Trm-like phenotype was enhanced in individuals with a history of smoking [46]. In gastrointestinal cancers, they actively survey the TME, equipped to detect malignant cells and initiate immune responses [30]. This proactive surveillance by Trm cells is integral to our understanding of their contribution to antitumor immunity.

### 2.2. Trm Cells as Prognostic Factors

Studies have indicated a significant influence of Trm cells on tumor progression and patient outcomes in pancreatic cancer and CRC. Trm cells in pancreatic ductal adenocarcinoma co-express anti-programmed cell death protein 1 (PD-1) and TIGIT (T Cell Immunoreceptor with Ig And ITIM Domains). Moreover, the combination of anti-PD-1 and TIGIT inhibition therapy enhances IFN-γ secretion and T-cell proliferation in the presence of PD-1 and TIGIT ligands [42]. With further study, it has become apparent that the abundance of Trm cells is a vital determinant of patient survival. In pancreatic cancer patients, those with a higher abundance of Trm cells tended to survive longer compared with those with lower numbers [47]. In left-sided CRC, the presence of activated Trm cells (not CD8 alone) was prognostically significant, and patients with low numbers of activated Trm cells exhibited a poor prognosis, even with high CD8 T-cell infiltration. Interestingly, patients with high CD8 T-cell infiltration and low numbers of activated Trm cells showed good prognosis in right-sided CRC [48].

Tumor invasion by Trm cells correlates with an increased response to current immunotherapies and is often associated with a favorable outcome [49]. Recent studies analyzing Trm cells from the intrinsic layer and epithelial compartment of the small intestine and colon have revealed molecular heterogeneity and a varying dependence on EOMES, which warrants further study [50]. Trm cell formation and maintenance are influenced by several factors, including inflammation, antigen induction, and tissue-specific cues, which suggests that these signals also contribute to heterogeneity within the Trm cell compartment [51].

Although not previously reported in gastrointestinal cancers, CD8+CD103+ tumor-infiltrating lymphocytes are memory T cells that reside in tumor-specific tissues and are considered a prognostic factor for survival in lung cancer patients [44]. The number of CD103+/CD8+ tumor-infiltrating lymphocytes (TILs) is a good prognostic and predictive factor for overall and relapse-free survival in patients with CRC [43]. Based on a combined analysis of single-cell and bulk RNA-sequencing data, the Trm-related gene risk score was closely correlated with the prognosis and treatment response in patients with CRC [52]. The percentage of resident memory CD103-expressing CD8+ and γδTCR+ intraepithelial lymphocytes was markedly reduced in the left and right colon of patients with familial adenomatous polyposis compared with healthy controls [53]. In esophageal cancer, patients with CD103^high^ biopsy specimens showed a favorable prognosis, whereas chemotherapy increased the number of CD103+ cells [54]. The presence of CD103+CD8+ TILs, a Trm cell phenotype, and high expression of immune checkpoints PD-1 and TIM-3 in esophageal squamous cell carcinoma (ESCC) was positively correlated with the overall survival of ESCC patients.

This suggests that this cell population may induce strong proliferation, cytotoxic cytokine production, and antitumor immunity following anti-PD-1 inhibition [55]. Immune checkpoint inhibitors (ICIs) have revolutionized cancer therapy, and the high frequency of CD103 in anti-PD-1+CD8+ T cells two weeks after nivolumab administration in patients with advanced gastric cancer (GC) may be a useful biomarker for predicting the efficacy of anti-PD-1 therapy [56]. In GC, low levels of tumor-infiltrating CD8+CD103+ Trm cells were positively correlated with GC progression and decreased patient survival [57]. CD103+ T cells are located around tertiary lymphoid structures (TLSs), and TLSs are more abundant in CD103^high^ patients [58].

Furthermore, ZNF683 expression was identified as a candidate biomarker of cancer-specific Trm cells and a promising target for cancer immune regulation [43,59]. Some CD8+ T cells derived from colorectal liver metastases preferentially re-populate patient-derived autologous xenograft tumors as Trm cells [60]. In esophageal cancer, specimens rich in Trm cells showed reduced lymphatic invasion and lymph node metastases as well as prolonged survival compared with specimens with fewer Trm cells [61].

Changes in the gastric microbiome are linked to a decrease in CD8+ Trm cells in the TME of GC [62]. An analysis of the role of Trm cells in enhancing immunogenicity in CRC stratified by microsatellite instability (MSI) and BRAF status revealed that in both BRAF mutants and MSI-H BRAF wild-type MSI-H CRCs, CD8+ Trm cells were more abundant compared with the microsatellite stable group [63]. Ovarian cancer is an immunogenic disease that is dependent on approximately 13% of CD8+ tumor-infiltrating T cells that are initially stimulated against high-affinity antigens and maintain a wave of effector Trm-like cells [64], which was also found to be potentially applicable to other tumors.

Recent studies have revealed an intricate link between the abundance of Trm cells within the TME and favorable survival outcomes [65,66,67,68,69,70,71,72,73]. They have yielded valuable insight into the prognostic implications of Trm cell infiltration, which further underscores their significance in gastrointestinal malignancies.

### 2.3. Trm Cells in Hepatocellular Carcinoma and Other Liver Diseases

In hepatocellular carcinoma (HCC), patients with predominantly exhausted CD8+ T cells (TEX) showed lower survival rates compared with those with a predominance of Trm cells. These T-cell populations were considered important novel biomarkers [65,66]. The expansion of peripheral Vγ9Vδ2 T cells with aminobisphosphonate reproduces the Trm cell phenotype and increases their cytotoxic potential. When combined with intratumor delivery, they may achieve more effective HCC immunotherapy [74].

One study elucidated the mechanism through which Trm cells coordinate the enhancement of viral clearance by potentiating local lymphoid sites [75]. Because of the potent effector function of hepatic Trm cells, they are essential not only for HCC but also for chronic liver diseases, including viral and parasitic infections, autoimmune liver diseases, non-alcoholic fatty liver disease, and liver transplantation. Moreover, the manipulation of hepatic Trm cells offers a new strategy for precision immunotherapy of chronic liver diseases [76]. Single-cell transcriptome and fluorescence-activated cell sorting analyses revealed enriched CD69+CD103−CD8+ Trm cells in NASH resolution livers [77]. Further insight into intrahepatic Trm cells will lead to a better understanding of the pathophysiology of many liver diseases and the identification of potential drug targets for the development of new therapeutics [78].

## 3. Trm Cells and Therapeutic Implications

Trm cells have become an important player in the immune response against cancer, particularly within the complex milieu of gastrointestinal tumors, such as pancreatic cancer and CRC. The discovery of Trm cells within these malignancies has sparked considerable interest in their potential use in therapeutic applications. Intratumor Trm cells express checkpoint inhibitory receptors, such as PD-1 and LAG-3; however, their induction can cause dysfunction, often referred to as fatigue, which may limit the effectiveness of Trm cells in countering tumor growth [79].

### 3.1. Trm Cells in Gastrointestinal Tumors and Immunotherapy

Intestinal tissue is often a target for the local growth of pathogens and invasion prior to entering the systemic circulation. It is also a prominent site of tumorigenesis; thus, promoting Trm cell formation at this site represents an attractive therapeutic option [80]. In this section, we explore how harnessing Trm cells can transform the clinical management of gastrointestinal cancers and other multiple carcinomas, including their relationship with ICIs [81].

**Figure 3:**
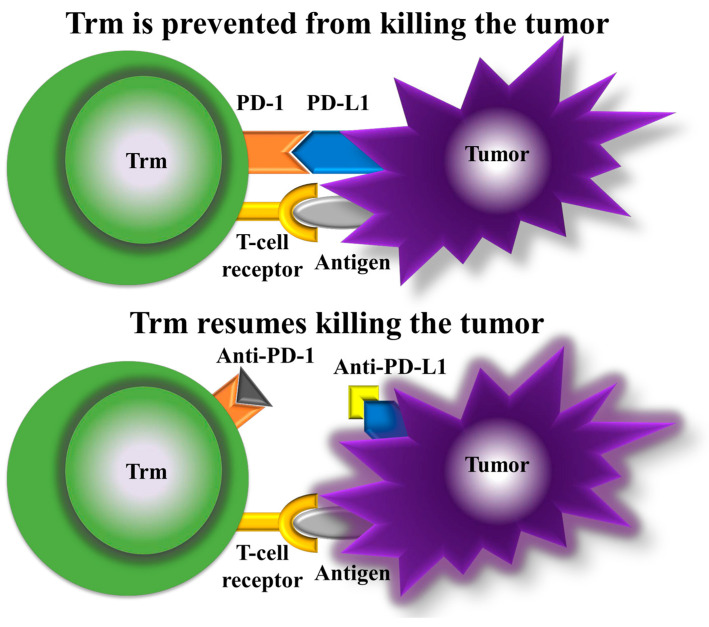
Mechanism of action of anti-PD-1 and anti-PD-L1. With PD-1 and PD-L1 bound (**top panel**), Trm cells are prevented from killing the tumor, but with anti-PD-1 and anti-PD-L1 (**bottom panel**), Trm cells resume tumor killing.

Immunotherapy has ushered in a new era of cancer treatment. The inclusion of Trm cells as a therapeutic target adds an intriguing dimension. PD-1 blockade promotes the proliferation of highly suppressive PD-1+ eTreg cells in hyperprogressive disease (HPD), which results in the inhibition of antitumor immunity and is a reliable marker of HPD. Moreover, the depletion of effector Treg (eTreg) cells in tumor tissues is effective for the treatment and prevention of HPD in PD-1 blockade cancer immunotherapy [81] (Figure 3).

### 3.2. Neoadjuvant Therapies and Clinical Outcomes of ICIs

Neoadjuvant anti-programmed death protein-1 (PD-1) or anti-PD-1/cytotoxic T-lymphocyte antigen-4 (CTLA-4) therapies in patients with oral cancer exhibit promising clinical activity [82]. The analysis of samples from a phase II clinical trial of head and neck oral squamous carcinoma patients who received neoadjuvant immune checkpoint blockade (ICB) therapy revealed an association of PD-1+/KLRG1- CD8+ T cells with pathologic response, which supports its use as a potential biomarker of ICB response [83].

ICIs targeting PD-1 and CTLA-4 significantly improve the outcomes of metastatic melanoma patients and reduce relapse in resected stage III disease [84]. In patients with metastatic vaginal melanoma, Trm cells are located in the tumor periphery. Trm cells exhibited an excellent functional response to autologous tumor cells and predicted neoantigens and melanoma differentiation antigens, with CD8+ Trm cells showing the highest tumor responsiveness. This suggests that Trm cells retain a strong antitumor T-cell functional response, which further increases following anti-PD-1 therapy [85].

### 3.3. Enhancing Trm Cell Function and Prognosis in Various Cancers

In mice with CRC liver metastases, the inhibition of the renin–angiotensin system reduced the abundance of immunosuppressive bone-marrow-derived suppressor cells and enhanced PD-1+ hepatic Trm cells, which suggests that it may be an effective adjuvant therapy for patients with CRC liver metastases [86]. ICIs, including nivolumab, have been approved for the treatment of esophageal cancer, and patients with an abundance of Trm cells have a better prognosis after starting nivolumab therapy. This suggests that Trm cells are important prognostic factors [87]. The prophylactic use of ICIs during the early stages of ESCC potentially provides long-term benefits to patients [88]. Thus, these CD103+CD8+ Trm cells may be regulated by retinoic acid metabolism [89]. These cells are pivotal in enhancing the effectiveness of ICIs, thereby expanding their utility in clinical practice. In addition, the development of Trm-cell-based therapies has the potential to provide novel treatments that enhance patient outcomes [90].

### 3.4. Trm Cells and Immune-Related Adverse Events

Moreover, our understanding of the etiology of ICI-induced colitis has improved. CD8+ Trm cell activation correlates with the severity of clinical and endoscopic ICI colitis, and activated CD8+ Trm cells express high levels of checkpoint inhibitors and IFN-γ transcripts in ICI colitis [90]. TCR-sequencing analysis identified cytokines, chemokines, and surface receptors that may serve as therapeutic targets for inflammatory side effects [91]. In addition, immune-related adverse events (irAEs), which are frequently caused by ICIs and can be life-threatening in some cases, as well as the expression of IFNγ, CXCL9, CXCL10, and TNFα in irAE dermatitis, have been confirmed, particularly in Trm cells [92].

### 3.5. The Impact of IL Pathways in Trm-Cell-Mediated Cancer Therapy and Psoriasis Treatment

The interaction between Trm cells and ICIs is a focal point in the evolving landscape of cancer therapy. Trm cells act as sentinel cells within the TME to promote an immune response against cancer cells when checkpoint inhibitors are administered. This interplay suggests the potential for synergy and improved therapeutic outcomes. Clonotype and trajectory analyses of the TME in GC revealed that Tc17 cells (IL-17 + CD8 + T cells) are derived from Trm cells and can subsequently differentiate into depleted T cells. This suggests the possibility of targeting IL17+ cells and associated signaling pathways as a therapeutic strategy for the treatment of GC [93]. In psoriasis, IL-17A-producing CD8+ Trm cells represent an attractive therapeutic target because they are considered one of the pathogenic populations in the skin [94]. Trm cells are classified as CD8+ Trm cells and are primarily distributed in the epidermis, whereas CD4+ Trm cells reside in the dermis. CD8+ Trm cells are derived from circulating memory T cells, and CD49a-CD8+ Trm cells have an important role in the recurrence of psoriasis because IL-23 can also activate Trm cells. Thus, neutralizing antibodies against IL-23 may be effective in the clinic [95].

### 3.6. Trm Cells in Infection, Immunity, and Prevention

In addition to therapy, a neoantigen peptide vaccine was developed for HCC against Trm cells. When combined with α-PD-1, it significantly increases the number of CD8+ Trm cells and exhibits potent tumor-killing ability [96]. In addition, the newly reported CD4+ Trm cells may be important for advancing the design of new vaccines and the development of new therapies for CD4+ T-cell-mediated autoimmune diseases [97].

**Figure 4:**
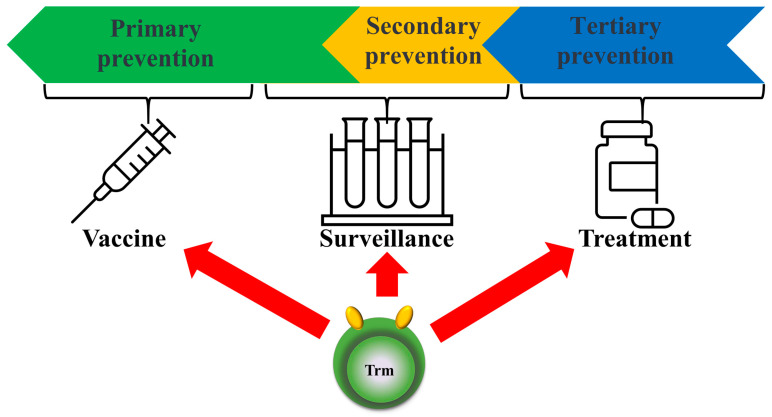
Prospects for future clinical applications of Trm cells. The concept of Trm cells is not only used for treatment (tertiary prevention) but also surveillance for primary/secondary prevention and for vaccines (primary prevention).

Applying our knowledge of local Trm cell function not only enables tertiary prevention, such as the early treatment of tumors, but also plays a major role in secondary prevention, such as immune surveillance, particularly for early cancer detection [98]. Although Trm cells are key cell types involved in the early detection and restriction of mucosal pathogens, following tissue-specific infection or vaccination, they remain in the tissue and perform rapid sensing and alarm functions to control reinfection by various respiratory pathogens, such as influenza and respiratory syncytial agents [99]. Herpes simplex virus and cytomegalovirus infections in both mice and humans constitute particularly important viral sanctuaries and induce Trm cells in mucosal tissue, which is the site of entry associated with attack and overlapping infection [100]. Examination of CD4+ and CD8+ Trm cells in the lungs of BALB/c mice after acute respiratory syncytial virus (RSV) infection revealed enhanced viral clearance. This suggests that CD8+ Trm cells contribute to protection against secondary RSV infection, and given this protective capacity, they are considered a future RSV vaccine candidate [101]. Further understanding of the mechanisms of protective immune enhancement will improve rational vaccine development for primary prevention [96,102,103] (Figure 4).

### 3.7. Future Directions and Therapeutic Potential

The multifaceted understanding and application of Trm cells could be a major contributor to cancer therapy and the treatment of infectious and autoimmune diseases. A summary of the references that we have discussed regarding Trm cells in various cancers is listed in Table 1. As we explore the therapeutic implications of Trm cells in gastrointestinal cancers, the results indicate that these tissue-resident immune cells have the potential to revolutionize the treatment of patients with various types of cancers. Innovative treatment strategies that exploit Trm cells and their synergy with checkpoint inhibitors will lead to more effective and personalized cancer therapies.

## 4. Conclusions

In conclusion, the role of tissue-resident memory T (Trm) cells in gastrointestinal (GI) cancers, particularly pancreatic cancer and colorectal cancer (CRC), represents a promising frontier in cancer immunotherapy. The distinct ability of Trm cells to reside in nonlymphoid tissues and provide rapid, localized immune responses underscores their potential as pivotal players in the fight against GI malignancies. Recent studies have demonstrated that Trm cells in the tumor microenvironment (TME) of GI cancers can significantly influence tumor progression, patient outcomes, and responses to immunotherapy.

The presence of Trm cells within pancreatic cancer and CRC tissues has been correlated with improved prognosis and survival rates [42,47]. These cells exhibit unique phenotypic and functional characteristics that enable them to perform robust immune surveillance and mount effective antitumor responses [30]. For instance, the co-expression of inhibitory receptors such as PD-1 and TIGIT on Trm cells in pancreatic cancer highlights the potential of combined inhibition therapies to enhance antitumor immunity [42]. Moreover, the density and activation state of Trm cells in CRC have been linked to differential prognostic outcomes depending on the tumor location, further emphasizing their role as critical determinants of patient survival [48].

Trm cells also show promise as biomarkers for predicting the efficacy of immune checkpoint inhibitors (ICIs), such as PD-1 and CTLA-4 blockers. Patients with a higher abundance of Trm cells often exhibit better responses to ICIs and improved clinical outcomes [43,56]. This highlights the therapeutic potential of targeting Trm cells to enhance the effectiveness of current immunotherapy strategies. Additionally, the modulation of Trm cell activity through various adjuvant therapies, such as renin–angiotensin system inhibitors, has shown potential in augmenting the antitumor responses in CRC liver metastases [86].

As research advances, the therapeutic manipulation of Trm cells could revolutionize the treatment landscape for GI cancers. Developing strategies to increase the infiltration and activation of Trm cells within the TME, while mitigating the risk of immune-related adverse events, will be crucial. Furthermore, the integration of Trm-cell-targeted therapies with existing modalities, including ICIs and personalized vaccine approaches, offers a promising pathway toward more effective and durable cancer treatments.

In summary, the emerging insights into the role of Trm cells in GI cancers underscore their potential as powerful mediators of antitumor immunity. By leveraging their unique properties and understanding the mechanisms that regulate their function, we can pave the way for innovative and personalized therapeutic strategies that improve patient outcomes and advance the field of cancer immunotherapy.

## Figures and Tables

**Table 1:** Summary of references for Trm cells in various cancers.

Cancer Type	Reference	Main Findings
Pancreatic Cancer	[42,49]	Trm cells co-expressing anti-PD-1 and TIGIT correlate with improved outcomes; tumor invasion of Trm cells is associated with increased response to immunotherapy.
Colorectal Cancer (CRC)	[43,48,52,58,61]	Presence of CD8+CD103+ Trm cells is prognostically important; abundance correlates with patient survival; Trm-related gene risk scores are closely correlated with prognosis; CD8+ T cells derived from colorectal liver metastases preferentially re-populate tumors as Trm cells; CD8+ Trm cells are more abundant in BRAF mutants and MSI-H CRCs; a decrease in CD8+ Trm cells is associated with changes in gastric microbiome.
Esophageal Cancer	[54,55,87,88]	The presence of CD103+CD8+ TILs correlates with favorable prognosis; Trm cells may induce strong antitumor immunity following anti-PD-1 inhibition; Trm cells are important prognostic factors; prophylactic use of ICIs is potentially beneficial; CD103+ T cells are located around tertiary lymphoid structures; CD103^high^ biopsy specimens are associated with chemotherapy and improved survival.
Gastric Cancer (GC)	[56,73,93]	High frequency of CD103 in anti-PD-1+CD8+ T cells may predict efficacy of anti-PD-1 therapy; Tc17 is derived from Trm cells; IL17+ cells are potential therapeutic targets; clonotype and trajectory analyses reveal Tc17 lineage from Trm cells; CD103+CD8+ Trm cells are enriched in the tumor periphery; a potential therapeutic strategy targets IL-17+ cells and associated signaling pathways.
Liver Cancer (HCC)	[63,64,72,76,96]	Abundance of Trm cells correlates with improved survival; aminobisphosphonate-based expansion enhances Trm cell cytotoxic potential; Trm cells are essential for chronic liver diseases; neoantigen peptide vaccine against Trm cells shows promise; inhibition of the renin–angiotensin system enhances hepatic Trm cells; CD69+CD103−CD8+ Trm cells are enriched in NASH resolution livers; Trm cells are potential therapeutic targets for precision immunotherapy of chronic liver diseases.
Oral Cancer	[82,83]	Neoadjuvant immune checkpoint blockade therapy shows promising clinical activity; PD-1+/KLRG1- CD8+ T cells are associated with pathologic response; PD-1 and CTLA-4 therapies are potentially beneficial; CD8+ Trm cells demonstrate strong functional response to autologous tumor cells; CD103+CD8+ T cells are an important prognostic factor for survival.

## Data Availability

The data that support the findings of this study are available from the corresponding author upon reasonable request.
